# Cardiopulmonary endurance-training responsiveness of metabolic syndrome patients to individualized and standardized exercise prescriptions: a randomized controlled trial

**DOI:** 10.3389/fphys.2025.1427629

**Published:** 2025-03-14

**Authors:** Ruojiang Liu, Jinmei Qin, Xiang Zhang, Feng Wang, Weizhen Xue

**Affiliations:** ^1^ Physical Education College, North University of China, Taiyuan, China; ^2^ Heart Rehabilitation Center, Peking University First Hospital Taiyuan Hospital, Taiyuan, China; ^3^ The Ninth Clinical Medical College of Shanxi Medical University, Taiyuan, China

**Keywords:** ventilatory threshold, maximum physiological value, cardiopulmonary exercise test, cardiopulmonary endurance, metabolic syndrome

## Abstract

**Objective:**

This investigation compares the effects of two exercise prescriptions with equal energy consumption but different exercise intensity-determination methods on cardiopulmonary endurance in a population with metabolic syndrome (MetS). This investigation verified the effectiveness of individualized methods in patients with MetS undergoing moderate-intensity exercises.

**Methods:**

The participants were randomized into a standardized group or individualized group. Exercise intensity was determined based on the heart rate reserve method in the standardized group and ventilatory threshold model in the individualized group. The two groups completed 12 weeks of an exercise prescription with equal exercise frequency and energy consumption. Using cardiopulmonary exercise testing (CPET), primary and secondary cardiovascular endurance indicators were measured. The percentage change of PeakVO_2_ was used to classify participants as responders and non-responders. Other markers were used in auxiliary analysis of individual training responses.

**Results:**

A total of 40 MetS participants (75% male; mean age: 43.58 ± 11.73; body mass index: 30.39 ± 4.26) completed all exercise interventions. The PeakVO_2_ increased significantly (P < 0.05) in both the standardized and individualized groups. Significant improvements in peak heart rate and maximum voluntary ventilation were observed in the individualized group. Differences in training responsiveness were also observed between the standardized and individualized groups, with 70% and 90%, respectively, being classified as responders, and improvements in PeakVO_2_ experienced by 14.6% and 22.1%, respectively. During the training period (weeks 4–12), a significant difference in responsiveness was observed between the groups. Similar adverse changes were present in the CPET markers of adverse responders.

**Conclusion:**

The ventilatory threshold model-based individualized method has advantages in the MetS population. However, the responsiveness to the individualized method did not reach 100% in patients with MetS.

## 1 Introduction

Metabolic syndrome is a group of complex metabolic disorders that includes central obesity, insulin resistance, hypertriglyceridemia, hypercholesterolemia, hypertension, and decreased high-density lipoprotein cholesterol concentrations. A definitive diagnosis of MetS can be obtained when three or more of the five major metabolic abnormalities are present (Alberti et al., 2009). All age groups and both sexes of the MetS population manifest a higher risk of mortality from coronary heart disease, cardiovascular disease, and all-cause mortality than the non-MetS population. Even without a medical history of coronary heart disease, the risk of cardiogenic sudden death is increased by 70% in the MetS population, and the cardiovascular effects of MetS will increase risk events two-fold or more (Tirandi et al., 2022). Exercise-induced cardiorespiratory fitness (CRF) is an index that protects against the aforementioned MetS risk factors. Peak oxygen uptake (PeakVO_2_) and ventilatory threshold oxygen uptake (VO_2_@VT1) are significantly lower in the MetS population than in the healthy population (Kim et al., 2020; Rodriguez et al., 2022). Currently, the efficacy and safety of various aerobic exercises in enhancing cardiometabolic health of this cohort have been widely acknowledged (Batrakoulis et al., 2022b; Batrakoulis et al., 2021), and they are gaining increasing popularity among patients as non-pharmacological intervention measures (A'Naja et al., 2024). However, a large number of studies have revealed that various populations with different physical conditions, such as healthy sedentary individuals, overweight or obese individuals, or postmenopausal women, all demonstrate great heterogeneity in the CRF benefits acquired after exercise training, producing a large number of non-responders (Skinner et al., 2001; Scharhag-Rosenberger et al., 2012; Mattioni Maturana et al., 2021). Many authors (Wolpern et al., 2015; Dalleck et al., 2016; Weatherwax et al., 2016; Weatherwax et al., 2018b; Weatherwax et al., 2019) have applied the ventilatory threshold model to sedentary populations to effectively improve training “non-responsiveness,” and maximal oxygen uptake (VO_2max_) produced a 100% exercise training-response rate in these individuals. The ventilatory threshold model training could thus induce more uniform physiological responses to exercise stimulation in individuals, and this is regarded to be more effective in reducing inter-individual metabolic differences than the traditional method of standardizing exercise intensity based on the relative percent of maximal physiologic values (Meyler et al., 2021).

Participants in previous investigations were not all patients clinically diagnosed with MetS, but merely included sedentary populations at high risk of MetS (Wolpern et al., 2015; Weatherwax et al., 2016; Dalleck et al., 2016; Weatherwax et al., 2018b; Weatherwax et al., 2019). The effectiveness of the ventilatory threshold model in the MetS population thus requires experimental evidence. Due to differences in MetS severity and disease progression, the MetS population includes patients who have already developed and recovered from cardiovascular events and patients who started taking drugs to decrease blood pressure. Complex pathological processes, drug effects, and generally declining cardiac function and exercise capacity make it difficult for patients with MetS to sustain exercise at or above the second ventilatory threshold (VT2) (Chen et al., 2022), so the intensity of the ventilatory threshold model used in the aforementioned study needs to be adjusted. Furthermore, patients with MetS often suffer from comorbidities such as chronic obstructive pulmonary disease, chronic heart failure, and mitochondrial dysfunction (Bhatti et al., 2017; Chan et al., 2019), which can lead to difficulties in determining VT2 and even an absence of VT2 (Binder et al., 2008).

To meet the needs of this specific clinical population in this study, we ensured that the operational rules of the cardiopulmonary exercise test (CPET) complied with existing cardiac rehabilitation guidelines and the Harbor-UCLA Medical Center standards (Hansen et al., 2022; Glaab and Taube, 2022). Investigators previously recommended that a verification test (VER) with a higher workload than that in the final stage of the maximal incremental test be performed within 1 week following the incremental test for the determination of the VO_2max_ of the participants. VERs can avoid confusion as to whether actual VO_2max_ changes before and after intervention were stimulated by exercise or other factors, including routine changes, individual exercise training experience, willpower, environmental stimulation, and psychological effects (Weatherwax et al., 2018a; Weatherwax et al., 2019; Poole and Jones, 2017). The participants in the present study were all MetS clinical patients, but due to limitations such as experimental costs and subject acceptance, VER was not conducted and many participants did not achieve a VO_2_ plateau during CPET. Due to experimental rigor, we also expressed individual VO_2max_ as PeakVO_2_. In existing cardiac rehabilitation guidelines (Hansen et al., 2022; Mezzani et al., 2013), commonly used definitions of maximal or close to maximal effort includes respiratory exchange ratio (RER) ≥ 1.10, appearance of VO_2_ or an HR plateau, and rate of perceived exertion (RPE) ≥ 18/20. Although PeakVO_2_ cannot replace VO_2max_ and may lead to underestimation in some MetS patients (Moreno-Cabañas et al., 2020), PeakVO_2_ is still an effective marker for evaluating the CRF level of patients (Green and Askew, 2018). In addition, many authors applied PeakVO_2_ to evaluate the risk of developing MetS (Kim et al., 2020; Rodriguez et al., 2022). There is currently a paucity of VO_2max_ verification protocols that are suitable for MetS-specific clinical cohorts (Astorino and Emma, 2021). Based on the above information, all participants in our study underwent low-intensity adaptive training with equal exercise capacity and intensity at Weeks 1–4 to avoid PeakVO_2_ measurement errors that are often caused by anxiety due to lack of exercise training experience and unfamiliarity with the exercise model in CPET (incremental power bicycle), and to ensure that all participants can achieve PeakRER ≥1.10. Studies have shown that the magnitude of the VO_2max_ increase due to exercises below VT1 intensity was far lower than with exercises at VT1-and-above intensity (Farah et al., 2014). Low-intensity exercise stimulation cannot effectively activate signaling pathways needed to increase VO_2max_ (Margaritelis et al., 2018; Bishop et al., 2019). Therefore, we hypothesized that Weeks 1–4 of low-intensity exercise would not affect CRF training in the subsequent 8 weeks of training.

In the present study, we sought to determine whether, with overall exercise intensity < VT2, the individualized exercise prescription with the exercise intensity determined based on a ventilatory threshold model resulted in greater improvements in CRF in the MetS population when compared with the standardized CRF exercise prescription with the exercise intensity determined based on the relative percent of maximal physiologic values.

## 2 Study materials and methods

### 2.1 Participant criteria and ethical approval

Individuals who were eager to enhance their physical health through exercise and were willing to provide medical examination reports and medical records underwent assessment by the physician at the cardiac rehabilitation center of the Peking University First Hospital Taiyuan Hospital to initially determine their suitability for participation in this study. These patients actively inquired about the study and came to the center after obtaining information through means such as seeing flyers and display boards in the hospital. The assessment prior to CPET comprises the verification of materials including physical examination reports and an oral interview to obtain basic demographic, lifestyle, and medical data. Patients who met all the inclusion criteria and none of the exclusion criteria (Table 1) were eligible to participate in CPET. These qualified patients were required to complete CPET measurements within the ensuing week. The physician further screened the patients’ CPET baseline data to complete the verification of all exclusion criteria (Table 1). After the formal initiation of the exercise intervention, patients meeting the following criteria were removed from the study: (1) poor compliance or non-completion of the expected 12-week exercise volume within 3 months; (2) an RER of baseline CPET <1.05 and an RER of CPET <1.10 after 4 weeks of adaptive training for various reasons; (3) presentation with elevated blood pressure, abnormal electrocardiogram, or other condition during the exercise intervention to the extent that the rehabilitation therapist could no longer fully ensure their safety for continued exercise; and (4) notification of withdrawal due to personal reasons.

**TABLE 1:** Detailed inclusion and exclusion criteria.

Inclusion criteria	Exclusion criteria
• Meet the Chinese Diabetic Society diagnostic criteria for MetS, which required meeting three or more of the following five items: (i) abdominal obesity (i.e., central obesity), defined as waist circumference ≥90 cm in males and ≥85 cm in females; (ii) hyperglycemia, defined as fasting blood glucose ≥6.1 mmol/L, blood glucose ≥7.8 mmol/L 2 h after glucose loading, or diagnosis of diabetes mellitus (iii) hypertension, defined as blood pressure ≥130/85 mmHg or diagnosis with hypertension (iv) fasting triglycerides (TG) ≥ 1.70 mmol/L (v) fasting high-density lipoprotein cholesterol (HDL-C) < 1.04 mmol/L • Be aged 25–65 years • Exhibit a long-term sedentary lifestyle and not perform moderate-intensity physical activity for at least 3 days/week of 30 min/day duration within 3 months • Be willing to participate and sign an informed consent form	• Be in an acute phase of various diseases or with organ failure, tumor, myocardial infarction, cardiac insufficiency, myocarditis, or chronic lung disease • Engage in long-term alcohol consumption and/or be unable to ensure the performance of regular lifestyle habits during the intervention period • Be unable to ensure the performance of the stipulated exercise protocol during the intervention • Have poorly controlled blood pressure and/or blood glucose (resting blood pressure >150/100 mmHg and/or blood glucose >16.8 mmol/L) • Have unstable medication use and may require medication adjustment during the intervention • Be unable to cycle continuously at 60 rpm according to the metronome during CPET. • Present with conditions such as significantly elevated blood pressure during exercise and abnormal electrocardiogram findings during the CPET process

This study was approved by the Ethics Committee of Peking University First Hospital Taiyuan Hospital (no. 2022026), and all patients signed the informed consent form before the trial began (ClinicalTrials.gov, NCT06379204).

### 2.2 Marker measurement and procedure

A medical grade analyzer (HNH-318, Omron, Osaka, Japan) was used to measure the height and weight of participants and the body mass index (BMI) was calculated. Participants avoided alcohol, coffee, and other stimuli that would affect CPET measurements 24 h before CPET; were sufficiently rested, and avoided strenuous exhaustion or exercise. The weight, height, BMI, age, and sex of participants were inputted into the CPET operating system (CPX-770, Heart Gym, Beijing, China). Gas-volume calibration was conducted based on the manufacturer’s instructions and ambient temperature was stabilized at 19°C–21°C. Before the official test, participants sat quietly on a chair for 5 min with their back on the back rest, with both feet on the ground and palms supported at the heart level. An automatic upper arm sphygmomanometer (M5 professional, Omron, Mannheim, Germany) was used to measure resting heart rate and blood pressure. Resting pulmonary function was subsequently measured using a spirometer while the participant was in a seated position, thereby obtaining the baseline data of forced vital capacity (FVC) and maximum voluntary ventilation (MVV). A 12-lead electrocardiography system (EC-12S, Labtech, Debrecen, Hungary) and a blood pressure analyzer (Tango-M2, Suntech Medical, Miami, United States) were then connected to the participant. Participants rested for 3 min on a power bicycle (ergoselect100, Ergoline, Germany) and then cycled 3 min with no load warm-up at 60 rpm. The suitable incremental power was obtained by appropriately increasing or decreasing the predicted value generated by the operating system based on the participant’s weight, age, and sex, in light of the actual physical fitness level reported by the patient verbally. It enabled the participant to achieve symptom-limited maximal cardiopulmonary exercise testing within 6–10 min, which was followed by 5–10 min of recovery and end of the trial. During the official test, a qualified rehabilitation therapist closely watched the screen to monitor indicators such as oxygen intake, carbon dioxide equivalent, and ventilation rate in real-time. Based on the instructions of the physician, who monitored the participant’s dynamic electrocardiogram and exercise blood pressure in real-time, the therapist encouraged the patient throughout the process and assisted them in safely recovering when termination indications occurred. All participants were encouraged to deliver their maximal effort (Glaab and Taube, 2022; Mezzani et al., 2013; Hansen et al., 2022). Three methods were used to jointly determine the VT1: i.e., (1) the V-slope method, showing the slope inflection point of VCO_2_ vs VO_2_ relative increase during exercise; (2) the O_2_ ventilatory equivalent method (VE/VO_2_)—i.e., after incremental power had started, O_2_ utilization efficiency increased to its maximum while O_2_ ventilatory equivalent was at its lowest; and (3) end-expiration O_2_ partial pressure method—i.e., the lowest end-tidal alveolar O_2_ partial pressure. Although the system automatically calibrates VT1 after the test, the result is usually inaccurate. The therapist and physician responsible for the test used these three methods to manually adjust the VT1 value through visual inspection. To ensure that the measured values of PeakVO_2_ at baseline, at 4 weeks, and at the end of 12 weeks were not influenced by the confounding factor of test time, the testing of each patient at each stage was performed within the same time period of the week.

### 2.3 Exercise prescription formulation and implementation

**FIGURE 1:**
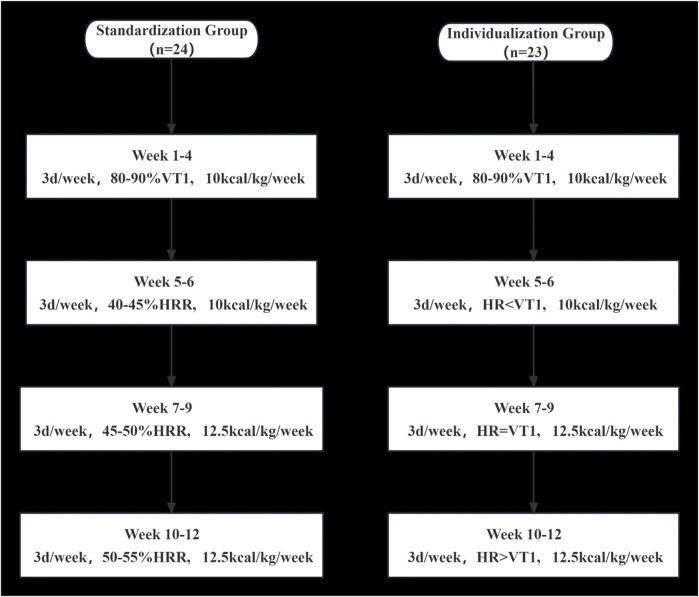
Details of exercise prescriptions at each stage from baseline to week 12 for the standardized group and the individualized group.

After baseline tests were completed, a simple random number sequence generated by SPSS 27.0 software was used to assign eligible patients to either the individualized group or the standardized group. Only the principal researcher responsible for interpreting the results of the trial and the therapists who prescribed the exercise regimens knew the group assignments of the participants. However, they were not involved in the implementation of the training sessions. The therapists responsible for the exercise intervention did not know the group assignments, and the participants did not know to which group they were assigned. Weeks 1–4 entailed the adaptive training stage. After 4 weeks, we conducted the CPET, and this was used to formulate the exercise prescription for the next 8 weeks. In the adaptive training of Weeks 1–4, the ventilatory threshold model was used to determine 80–90%VT1 exercise intensity for both groups. During Weeks 5–12, different exercise intensity-determination methods were used in the two groups, and exercise intensity gradually increased from moderately low to moderately high. The exercise volume was the same in the two groups: 10 kcal/kg/week in Weeks 1–6 and 12.5 kcal/kg/week in Weeks 7–12, and exercise frequency was 3 d/week and carried out on alternate days. Each patient exercised on a power bicycle that could be connected to a remote heart rate monitor. The exercise intensity-determination method for the standardized group was based on the heart rate reserve method (%HRR) of the American College of Sports Medicine (Liguori and Medicine, 2020), while exercise intensity in the individualized group was determined based on the ventilatory threshold model (Figure 1). The target exercise heart rate for the individualized group was calculated using the following method:

Weeks 1–4 (HR < VT1), the target HR range was higher than resting HR and lower than VT1—i.e., 90% VT1 > HR > 80% VT1;
Weeks 5–6 (HR < VT1), the target HR ranged from 10 bpm below VT1 to VT1—i.e. VT1 > HR > VT1-10 bpm;
Weeks 7–9 (HR = VT1), the target HR ranged from 5 bpm above VT1 to 5 bpm below VT1— i.e., VT1+5 bpm > HR > VT1-5 bpm;
Weeks 10–12 (HR > VT1), the target HR range was 5 bpm to 15 bpm above VT1—i.e., VT1 + 15 bpm > HR > VT1 + 5 bpm.

The key points of the exercise-prescription formulation in this study included the estimation of heart rate and oxygen uptake corresponding to exercise intensity and energy consumption. The total weekly exercise duration for each patient was determined by jointly calculating the estimated energy consumption of the target heart rate range and the pre-set individual weekly exercise volume according to the formula kcal/kg/week × body weight and was allocated among the three training sessions in each week. The exercise prescription for each patient was calculated separately by two rehabilitation therapists based on the results of the CPET operating system record before verification.

The patients were allowed to choose on which day of the week they initially participated in the training session, followed by a 1-day rest before the next session. All training was completed in the hospital’s cardiac rehabilitation center. Before each formal training session, therapists guided the patients in warming up for approximately 10 min, and then assisted them in adjusting the height and handlebars of the power bicycle to ensure a comfortable posture. Subsequently, a remote heart rate monitor (tkecg-h01, Oriental Thai Wah, Beijing, China) was worn by each patient, enabling the therapists to monitor the patient’s heart rates during exercise and ensuring that the actual exercise heart rate remained within the target heart rate range. In the first training session, the therapist progressively adjusted the power of the bicycle ergometer to achieve the target heart rate. Thereafter, adjustments were made for every exercise session based on the patient’s condition and exercise prescription. The duration of the endurance training for the patients was individually calculated based on the methods described above. The power bicycle computer system recorded the exercise duration of the patients after the start. The adjustment and cool-down stages before and after the formal training were excluded in the total duration, which was supervised by the therapist. Finally, each patient was required to perform stretching exercises to minimize any discomfort that they felt after the training. The therapists who supervised the training created a training log for each patient, which was used to record the exercise heart rate stipulated in the exercise prescription, the actual exercise heart rate, the power bicycle wattage, and RPMs, *etc.*

### 2.4 Statistical analysis

SPSS 27.0 was used for statistical analysis. Quantitative data are expressed as mean ± standard deviation (mean ± SD) and normality was tested based on the normality plot of the residuals in the analysis-of-variance model. The residuals were considered to be normally distributed when the Shapiro-Wilk test result was not significant. Sample size was estimated based on the change in VO_2max_ as the main outcome variable. The means and standard deviations from a previous study were used, and the effect size for this research was calculated (Weatherwax et al., 2019). Assuming a power of 0.80 was required and the calculated effect size for the change in VO_2max_ was 1.046, it was determined that approximately 16 participants would be needed per group. It was assumed there would be an approximate 20% dropout rate, so the aim is to achieve at least 20 participants per group until the study finishes. For indicators with significant changes before and after intervention in each group, intergroup comparisons were conducted. The primary measurement indicator, PeakVO_2_, was analyzed using repeated measures ANOVA, incorporating multiple measurement results. This analysis factored in age, sex, height, weight, BMI, and baseline values as covariates, with the exercise intensity-determination method being the primary focus. The Bonferroni method was applied for multiple test corrections. Additionally, other secondary indicators were evaluated using ANCOVA to analyze the main effects of the group, where applicable.

As the gas collection analysis for CPET in this study was controlled by a computer system, the technical principles conformed to current clinical standards, and the experimental design stipulated that subject testing be carried out using the same machine at the same time period on the same day of the week, thus minimizing error. Twelve participants were randomly selected and two baseline tests were completed on two different days in the same week to determine the coefficient of variation (CV) of PeakVO_2_(Weatherwax et al., 2018a; Hopkins, 2000), which we found to be 4.8%. We chose the change in the percentage of PeakVO_2_ between baseline and Week 4, Week 4 and Week 12, and between baseline and Week 12 (i.e., post-intervention—baseline value)/baseline value, and recorded as %Δ. The participants were classified as follows: 1 = responder (Δ > 4.8%) and 0 = non-responder (Δ ≤ 4.8%). Thereafter, response and non-response were used as qualitative data, and these underwent continuity correction by chi-squared analysis (χ^2^ test) and employed for analysis of response at baseline–Week 4, Week 4–12, and baseline–Week 12 to observe changes in response rate.

## 3 Results

**FIGURE 2:**
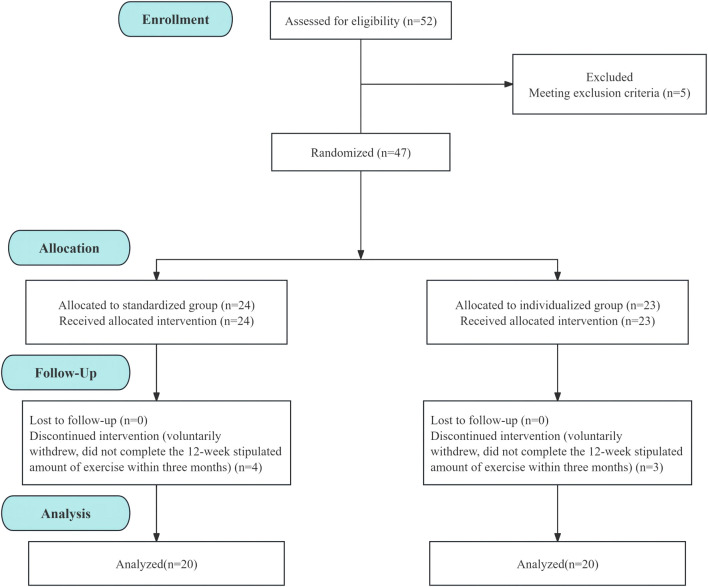
Baseline to Week 12 exercise intervention flow chart of the standardized and individualized groups.

Fifty-two participants were evaluated. During baseline CPET evaluation, one patient manifested high blood pressure during exercise, two experienced significant ST segment depression, and two showed unstable blood pressure, with drug modification scheduled. These participants were excluded from our study. We ultimately enrolled 47 eligible MetS patients. Among the 47 participants, seven withdrew during the intervention. The remaining patients demonstrated good tolerance to the exercise prescription and completed the 12-week stipulated amount of exercise within 3 months. The overall withdrawal rate was 14.9%. Three patients were removed from the individualized group (two had their intervention disrupted due to business trips and COVID-19 infection and one voluntarily withdrew), and four patients were removed from the standardized group (one could not complete the intervention due to lack of time and three voluntarily withdrew). The dropout rates for the individualized group and the standardized group were 13.0% and 16.7%, respectively. Finally, 40 patients (75% male; mean age: 43.58 ± 11.73; BMI: 30.39 ± 4.26) were included in the entire study (Figure 2). Table 2 depicts the basic information of the two groups at baseline.

**TABLE 2:** Basic demographic and clinical data of the standardized and individualized groups ( $\bar{x}$ ± *SD*).

	Standardized group (n = 20)	Individualized group (n = 20)	*P*
Age (years)	41.8 ± 10.55	45.35 ± 12.83	0.345
Sex (Male/Female)	16/4	14/6	0.715
Weight (kg)	93.49 ± 16.38	85 ± 15.59	0.101
Height (cm)	171.9 ± 7.85	170 ± 6.98	0.424
BMI (kg/m2)	31.54 ± 4.5	29.24 ± 3.77	0.088
Waist circumference (cm)	104.85 ± 9.68	98.05 ± 8.55	0.024
Body fat percentage	33.38 ± 6.09	33.14 ± 5.54	0.895
Triglycerides (mmol/L)	2.51 ± 1.34	2.42 ± 1.00	0.817
Total cholesterol (mmol/L)	5.20 ± 1.19	4.53 ± 1.12	0.077
Low-density lipoprotein (mmol/L)	3.46 ± 0.92	3.03 ± 0.83	0.127
High-density lipoprotein (mmol/L)	1.13 ± 0.18	1.00 ± 0.22	0.054
Systolic blood pressure (mmHg)	129.40 ± 10.15	126.95 ± 12.20	0.494
Diastolic blood pressure (mmHg)	85.90 ± 8.90	83.45 ± 10.13	0.422
Central obesity, n (%)	20 (100%)	18 (90%)	0.468
Hypertension, n (%)	13 (65%)	13 (65%)	1.000
Dyslipidemia, n (%)	18 (90%)	17 (85%)	1.000
Type 2 diabetes, n (%)	9 (45%)	11 (55%)	0.752
Statin, n (%)	5 (25%)	2 (10%)	0.405
Fibrates, n (%)	3 (15%)	2 (10%)	1.000
Oral hipoglycemiant, n (%)	7 (35%)	9 (45%)	0.747
Inhibitor of angiotensin-converting enzyme, n (%)	3 (15%)	0 (0%)	0.230
Angiotensin II receptor antagonists, n (%)	3 (15%)	0 (0%)	0.230
Beta-blocker, n (%)	2 (10%)	7 (35%)	0.130
Diuretic, n (%)	0 (0%)	2 (10%)	0.468
Calcium channel blockers, n (%)	6 (30%)	3 (15%)	0.449

### 3.1 CPET marker changes

After 12 weeks of exercise intervention, the PeakVO_2_, peak load power (PeakWR), peak metabolic equivalent (PeakMets), peak heart rate (HRpeak), peak oxygen pulse (PeakVO_2_/HR), peak respiratory exchange ratio (PeakRER), muscle work efficiency (ΔVO_2_/ΔWR), first ventilatory threshold (VO_2_@VT1), and MVV of MetS patients in the standardized group showed varying degrees of increase compared with before intervention, while oxygen uptake efficiency slope (OUES), minimum ventilatory equivalent for carbon dioxide (LowestVE/VCO_2_), slope of ventilatory equivalent for carbon dioxide (VE/VCO_2_slope), and FVC decreased slightly compared with before intervention, but only the differences in PeakVO_2_, PeakWR, PeakMets, PeakVO_2_/HR, and PeakRER were statistically significant (P < 0.05) (Table 3). Of these parameters, and relative to before intervention, PeakVO_2_ was increased by 14.6%, PeakWR was increased by 14.8%, PeakMets was increased by 12%, PeakVO_2_/HR was increased by 8%, and PeakRER was increased by 3.6%.

**TABLE 3:** CPET of standardized and individualized groups before and after intervention ( $\bar{x}$ ± *SD*).

Marker	Standardized group (n = 20)				Individualized group (n = 20)				Pre-intervention intergroup		Post-intervention intergroup	
	Before intervention	After intervention	*t*	*P*	Before intervention	After intervention	*t*	*P*	*t*	*P*	*t*	*P*
PeakVO_2_ (mL/min/kg)	25.12 ± 4.82	28.80 ± 5.91^*^	−3.600	0.002	21.92 ± 3.87#	26.77 ± 5.46*	−6.519	<0.001	2.317	0.026	1.128	0.266
PeakWR (watt)	168.75 ± 47.21	193.8 ± 52.59*	−5.089	<0.001	138.45 ± 49.04	161.6 ± 50.27*	−6.724	<0.001	1.991	0.054	1.979	0.055
PeakMets (met)	7.17 ± 1.37	8.03 ± 1.66^*^	−3.274	0.004	6.28 ± 1.11^#^	7.4 ± 1.56^*^	−5.885	<0.001	2.270	0.029	1.079	0.287
HRpeak (bpm)	158.25 ± 22.1	161.15 ± 18.64	−1.140	0.268	145 ± 23.99	150.9 ± 21.34^*^	−3.776	<0.001	1.817	0.077	1.618	0.114
PeakVO_2_/HR (ml/beat)	14.8 ± 3.18	15.98 ± 3.8^*^	−2.365	0.029	13.06 ± 3.39	14.34 ± 4.28^*^	−3.984	<0.001	1.714	0.095	1.285	0.207
PeakRER	1.12 ± 0.07	1.16 ± 0.05^*^	−2.570	0.019	1.11 ± 0.07	1.17 ± 0.06^*^	−6.851	<0.001	0.291	0.773	−0.537	0.595
OUES (mL/logL)	2675.85 ± 779.42	2569.68 ± 619.52	0.571	0.575	2273.61 ± 690.97	2277.83 ± 683.27	−0.073	0.943	1.727	0.092	1.415	0.165
LowestVE/VCO_2_	29.6 ± 2.35	29.01 ± 2.75	1.218	0.238	30.23 ± 2.57	29.89 ± 3.26	0.579	0.570	−0.803	0.427	−0.929	0.359
VE/VCO_2_ slope	27.15 ± 2.53	26.11 ± 2.2	1.734	0.099	26.76 ± 2.8	27.1 ± 2.22	−0.544	0.593	0.468	0.642	−1.404	0.168
ΔVO_2_/ΔWR (ml/min/Watt)	10.66 ± 0.75	10.73 ± 0.97	−0.279	0.784	10.43 ± 1.18	10.57 ± 0.97	−0.698	0.494	0.718	0.477	0.513	0.611
VO_2_@VT1 (mL/min/kg)	13.53 ± 2.3	13.96 ± 3.23	−0.672	0.510	12.83 ± 1.59	13.35 ± 2.29	−1.440	0.166	1.117	0.271	0.689	0.495
FVC (L)	4.2 ± 0.9	4.11 ± 0.84	1.227	0.235	3.57 ± 0.91#	3.69 ± 0.95	−2.044	0.055	2.198	0.034	1.473	0.149
MVV (L/min)	135.2 ± 26.26	137.13 ± 28.71	−0.398	0.695	120.42 ± 27.84	128.1 ± 32.42*	−2.716	0.014	1.728	0.092	0.933	0.357
Weight (kg)	93.48 ± 16.38	91.33 ± 15.64^*^	3.529	0.002	85.00 ± 15.59	81.96 ± 14.72^*^	4.563	<0.001	1.679	0.101	1.951	0.058
BMI (kg/m^2^)	31.54 ± 4.50	30.82 ± 4.38^*^	3.574	0.002	29.25 ± 3.76	28.20 ± 3.59^*^	4.682	<0.001	1.743	0.089	2.070	0.045

Values are mean ± SD., PeakVO_2_, peak oxygen uptake; PeakWR, peak load power; PeakMets, peak metabolic equivalent; HRpeak, peak heart rate; PeakVO_2_/HR, peak oxygen pulse; PeakRER, peak respiratory exchange ratio; OUES, oxygen uptake efficiency slope; LowestVE/VCO_2_, minimum ventilatory equivalent for carbon dioxide; VE/VCO_2_ slope, slope of ventilatory equivalent for carbon dioxide; ΔVO_2_/ΔWR, muscle work efficiency; VO_2_@VT1, first ventilatory threshold; FVC, force vital capacity; MVV, maximum voluntary ventilation.

*P < 0.05, significant intra-group difference before and after intervention;

#P < 0.05, significantly lower in the individualized group than in the standardized group before intervention.

In the individualized group, the PeakVO_2_, PeakWR, PeakMets, HRpeak, PeakVO_2_/HR, PeakRER, OUES, VE/VCO_2_slope, ΔVO_2_/ΔWR, VO_2_@VT1, FVC, and MVV of MetS patients showed varying degrees of increase compared with before intervention, while LowestVE/VCO_2_ declined compared with before intervention, but only the differences in PeakVO_2_, PeakWR, PeakMets, PeakVO_2_/HR, HRpeak, PeakRER, and MVV were statistically significant (P < 0.05) (Table 3). Of these parameters and compared with prior to intervention, PeakVO_2_ increased by 22.1%, PeakWR increased by 16.7%, PeakMets increased by 20.4%, HRpeak increased by 4.1%, PeakVO_2_/HR increased by 10%, PeakRER increased by 5.4%, and MVV increased by 6.4%.

Before exercise intervention, the FVC, PeakVO_2_, and PeakMets of the standardized group were significantly higher than the same indices in the individualized group (P < 0.05). However, after exercise intervention, there were no significant inter-group differences in the markers (P > 0.05) (Table 3). Even though these differences were not statistically significant, the individualized group showed elevations in the percentage changes in PeakVO_2_, PeakWR, PeakMets, PeakVO_2_/HR, and PeakRER that were greater than in the standardized group. The individualized group also experienced significant improvements in the HRpeak and MVV of MetS patients.

**FIGURE 3:**
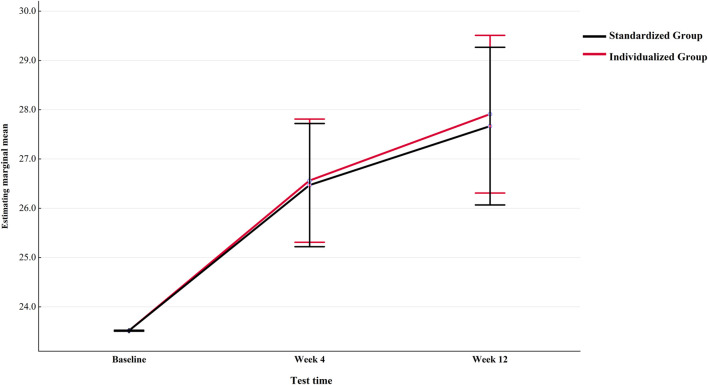
Trend of PeakVO_2_ changes in individualized and standardized groups from baseline to Week 12 (after correction).

The repeated measurement ANOVA corrected for age, sex, height, weight, BMI, and baseline values for the main measurement indicator PeakVO_2_ showed that the time main effect of the within-subject effect test was not significant, indicating that time was not a main factor causing changes in PeakVO_2_. Age and sex factors can affect the trainability of CRF (Table 4). PeakVO_2_ was comparable between the two groups at baseline to week 4. At 12 weeks, the individualized group tended to have higher PeakVO_2_ than the standardized group (Figure 3). This may suggest that if the intervention time is further increased, the inter-group differences may reach statistical significance. The ANCOVA results of other secondary indicators showed that after baseline correction, the group main effects were not significant (Table 5) for PeakMets, HRpeak, PeakVO_2_/HR, or MVV either.

**TABLE 4:** ANOVA results of repeated measurements of the main indicator PeakVO_2_ ( $\bar{x}$ ± *SD*).

Group	Baseline	Week 4	Week 12	*F*	*P*	η^2^
Standardized group	25.12 ± 4.82	27.87 ± 5.47	28.80 ± 5.91			
Personalized group	21.92 ± 3.87	25.16 ± 5.16	26.77 ± 5.46			
Group main effect				0.03	0.87	0.00
Time main effect				1.80	0.17	0.05
Time * Group				0.03	0.98	0.00
Baseline * Group				1.79	0.18	0.05
Gender * Group				4.27	0.02*	0.12
Age * Group				3.99	0.02*	0.11
Height * Group				2.03	0.14	0.06
Weight * Group				2.10	0.13	0.06
BMI * Group				1.80	0.17	0.05

Values are mean ± SD., PeakVO_2_, peak oxygen uptake. *P < 0.05, the interaction was statistically significant.

**TABLE 5:** Inter-group comparison of secondary indicators.

Response indicator	Corrected difference ( $\bar{x}$ ±*SD*)		Group effect		
	Standardized group	Individualized group	*F*	*P*	η^2^
PeakWR (watt)	25.15 ± 4.42	23.05 ± 4.42	0.12	0.74	0.003
PeakMets (MET)	0.99 ± 0.27	1.26 ± 0.27	0.57	0.46	0.02
HRpeak (bpm)	4.18 ± 2.00	4.62 ± 2.00	0.02	0.88	0.001
PeakVO_2_/HR (mL/beat)	1.09 ± 0.43	1.40 ± 0.43	0.24	0.63	0.01
MVV (L/min)	2.48 ± 4.07	7.12 ± 4.07	0.63	0.43	0.02

Values are mean ± SD., PeakWR, peak load power; PeakMets, peak metabolic equivalent; HRpeak, peak heart rate; PeakVO_2_/HR, peak oxygen pulse; MVV, maximum voluntary ventilation.

### 3.2 Prevalence of non-responders and responders

Table 6 depicts the prevalence of non-responders and responders in the standardized and individualized groups. After Weeks 1–4 of intervention, 65% (13/20) of participants in the standardized group and individualized group developed beneficial PeakVO_2_ changes (Δ > 4.8%), and Weeks 1–4 reflected 80%–90% VT1 exercise intensity. This indicated that PeakVO_2_ was significantly augmented in some participants under low exercise intensity. Some patients also exhibited significantly increased PeakVO_2_ due to a reduction in their lack of exercise experience during baseline testing, unfamiliarity with the exercise model, weak willpower, and other subjective factors.

**TABLE 6:** Chi-squared test results of response rates.

	Response	Group		Total	χ^2^	P-value
		Standardized group	Individualized group			
Baseline–Week 4	0	7 (35%)	7 (35%)	14 (35%)	0.000	1.000
	1	13 (65%)	13 (65%)	26 (65%)		
Total		20	20	40		
Week 4–Week 12	0	14 (70%)	4 (20%)	18 (45%)	8.182	0.004^*^
	1	6 (30%)	16 (80%)	22 (55%)		
Total		20	20	40		
Baseline–Week 12	0	6 (30%)	2 (10%)	8 (20%)	1.406	0.236
	1	14 (70%)	18 (90%)	32 (80%)		
Total		20	20	40		

*P < 0.05, a significant difference was present in the PeakVO_2_ response rate after intervention between the standardized group and individualized group.

**FIGURE 4:**
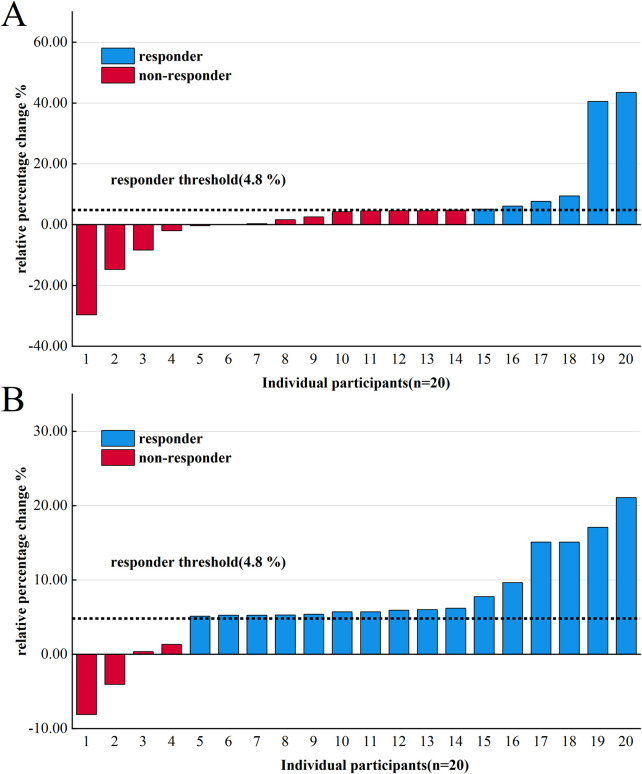
Variability of individual PeakVO_2_ following 4–12 weeks of endurance training in the standardized group **(A)** and the individualized group **(B)**.

From the end of Week 4 to Week 12, we adopted different methods of exercise intensity determination for the two groups. At the end of Week 12, compared with PeakVO_2_ at the end of Week 4, 30% (6/20) of participants in the standardized group were responders with beneficial changes (Δ > 4.8%), whereas 70% (14/20) of participants were non-responders with no meaningful changes in PeakVO_2_ (Δ ≤ 4.8%) (Figure 4A).

Conversely, in the individualized group, 80% (16/20) of participants were responders with beneficial changes in PeakVO_2_ (Δ > 4.8%), whereas 20% (4/20) of participants were non-responders with no meaningful changes (Δ ≤ 4.8%) (Figure 4B).

Overall observations from baseline to Week 12 indicate that the response rate in the individualized group was 90% (18/20) and the rate in the standardized group was 70% (14/20). χ^2^ analysis showed no significant difference in the baseline–Week 12 response rate (P = 0.236); however, a significant difference was observed in the response rates from Week four to Week 12 (P = 0.004) (Table 6).

## 4 Discussion

To our knowledge, this clinical trial was the first-ever to encompass the effects of two exercise prescriptions with equal energy consumption based on two exercise intensity-determination methods on cardiopulmonary endurance in the MetS population. In addition to employing PeakVO_2_ as the primary indicator of patients’ cardiopulmonary fitness, this research also applied submaximal secondary indicators of CPET for the analysis of the post-training improvement of CRF. The results demonstrate that when the individualized method is applied in patients with MetS, it can not only enhance the individual response rate of PeakVO_2_ but also significantly increase the MVV and HRpeak. However, within the clinical cohort of patients with MetS, the individualized method based on the ventilatory threshold model appears to be limited by specific factors.

In this study, the overall exercise intensity used in previous studies was diminished to accommodate patients with MetS, that is, the exercise intensity of VT2 and above was not used. This may be one of the reasons why there were no significant improvements in VO_2_@VT1, OUES, or ΔVO_2_/ΔWR markers (Gaskill et al., 2001; Guio de Prada et al., 2019). However, this study revealed that even moderate-intensity exercise could effectively increase PeakVO_2_, PeakWR, PeakMets, PeakVO_2_/HR, and PeakRER of the MetS population. At the group level, the group effect was not significant in the repeated measurement ANOVA of PeakVO_2_, and the individualized group only showed a trend of gradually increasing more than the standardized group after 4 weeks (Figure 3). However, at the individual level, only four patients (20%) in the individualized group had no meaningful change in PeakVO_2_ (Δ < 4.8%) after 4–12 weeks of exercise intervention, whereas 14 patients (70%) in the standardized group had no meaningful change (Table 6).

The baseline MVV accounted for a lower proportion of the estimate in some participants in the individualized group. After long-term, precise near-VT1 exercise training, passive inhalation in the patients changed to active inhalation to cope with gas exchange requirements during exercise. Near-VT1 intensity might have enabled participants to adjust respiration frequency easily and actively from shallow and rapid breathing to deep and slow breathing, which would have resulted in effective training of the diaphragm. After 12 weeks of exercise intervention, the significant elevation in MVV even exceeded expectations (Hackett, 2020). Furthermore, precise training near VT1 can reduce the time constant τ, which reflects the rate of oxygen mobilization and improve the efficiency of respiratory muscles in patients with MetS (Passoni et al., 2015).

The HRpeak increase may have related to damaged parasympathetic cardiac nervous regulation at baseline in MetS patients (and fasting blood glucose is the main influencing factor), and this was reflected in poorer heart rate dynamics. At the start of incremental load exercise in MetS patients, the heart’s ability to adjust the heart rate to cope with continuous increase in muscle O_2_ demand is reduced, affecting the exercise performance and HRpeak (Stuckey et al., 2014). Endurance training improves cardiac vagal tone, and increases heart rate variability (HRV) and baroreflex sensitivity (BRS) (Bhati et al., 2018; Pearson and Smart, 2018). In addition, the HRV of female patients with MetS is lower than that of healthy individuals (Stuckey et al., 2014). Therefore, the differences in the two groups may be caused by differences in MetS composition at baseline and inter-group sex factors. Exercise intensity is a major determining factor of the autonomic nervous system response, and sympathetic nervous activity is increased and vagal nerve activity is reduced during the rest-exercise transition. When exercise intensity reaches VT2 and above, vagal nerve activity is maintained at a low level while overall autonomic nervous system innervation declines (Sarmiento et al., 2013). At this point, vagal nerve activity is affected by mechanical traction on the sinoatrial node due to increased respiratory frequency and respiratory depth and not to nervous regulation (Cottin et al., 2004). Excessive ventilation also directly affects vagal nerve activity during exercise and suppresses cardiac autonomic nervous regulation (Pichon, 2007). Precise near-VT1 exercise stimulation can result in more long-term and uniform benign responses in the autonomic nervous system in MetS patients, while there may be an underestimation or overestimation of target intensity when maximal physiological value is used in participants in a standardized group (Jamnick et al., 2020; Iannetta et al., 2020). This error in intensity prescription may affect the responses to progressive increases in exercise intensity in the 12-week intervention. Whether the ventilatory threshold model-based individualized method possesses unique restorative effects on cardiac parasympathetic nervous regulation requires further validation.

Compared with previous investigations, our participants who received a ventilatory threshold-based individualized exercise prescription did not show a 100% response rate after training, and two women were non-responders and adverse responders after 12 weeks of exercise intervention, with changes of −3.51% and −12.56%. Analysis of other CPET markers of these two patients revealed that their PeakRER and PeakWR were higher than baseline values after 12 weeks of intervention, showing that the adverse response of PeakVO_2_ was not related to the effort put in by the participants. In these two patients, OUES, VO_2_@VT1, ΔVO_2_/ΔWR, and PeakVO_2_/HR fell and VE/VCO_2_slope and LowestVE/VCO_2_ significantly rose, indicating that their oxygen uptake capacity, exercise endurance, and ventilation efficiency had declined. These results may have been due to an increase in alveolar dead space, decreased pulmonary blood flow, diminished capacity for lung blood oxygenation, a mismatch between ventilation and perfusion, or mitochondrial dysfunction. It is still unknown whether such alterations are due to MetS progression, such as hyperglycemia, which seriously impairs aerobic remodeling of skeletal muscles, influencing the transport and utilization of oxygen (MacDonald et al., 2020). The gradual evolution of various components of MetS over time leads to pulmonary vascular remodeling and pulmonary hypertension (Willson et al., 2019). In the standardized group, one female patient showed the same adverse condition. Although the causes of adverse responses after exercise training cannot currently be explained, indirect analysis of CPET markers may constitute a solution. Additionally, previous studies have shown that long-term use of statins by MetS patients reduces their mitochondrial oxidative capacity, weakening the increase in PeakVO_2_ after aerobic exercise (Morales-Palomo et al., 2020). Although not all three patients were taking statins, it cannot be ruled out that there may be unknown drug effects interfering with the post-training response of PeakVO_2_.

In this study we observed that the overall CRF level of the MetS population was low. Compared with the VO_2_@VT1 (16.2 ± 4.1) of the MetS population previously reported (Rodriguez et al., 2022), the VO_2_@VT1 (13.18 ± 1.99) of MetS patients in this study was even lower. In addition, as the heart rate dynamics of the aforementioned MetS population were poor, the corresponding linear relationship between HR and VO_2_ was weakened (Stuckey et al., 2014; Silva et al., 2017). These changes all resulted in lower VT1-confirmed exercise heart rate, and the absolute exercise intensity in exercise intervention was lower than in the standardized heart rate reserve group. However, in the healthy populations of previous studies, the ventilatory threshold model confirmed that healthy exercise was higher than in the standardized method and patients exercised at a higher absolute intensity. These were all considered to be the direct results of using the ventilatory threshold model to determine exercise intensity (Weatherwax et al., 2019), and the uniqueness of the MetS population decreased this advantage. Thus, relying solely on heart rate monitoring for exercise intensity may not be suitable for patients with MetS.

For patients with heart disease, the range of %HRR corresponding to VT1 varies widely among individuals (beyond 40%–70% HRR) (Pymer et al., 2020). For some patients, the exercise prescriptions formulated based on %HRR far exceed their VT1, resulting in discomfort and excessive challenge during exercise, whereas others do not receive sufficient training stimulus to improve CRF. This study identified a similar situation among patients with MetS. This variation, coupled with the influences of type 2 diabetes, obesity, and the use of related medications, make it necessary to individualize the exercise prescription for MetS. Moreover, clinicians should select the corresponding VT intensity based on HR or workload according to the specific condition of the patient (Hansen et al., 2012). The higher the exercise intensity, the greater the training benefits. However, not using a gradual process and directly imposing high-intensity exercise (≥VT2) on patients may significantly increase their risk of acute myocardial infarction and sudden death (Bärtsch, 1999; Cadroy et al., 2002). After patients with MetS have achieved a relatively high level of physical fitness, it is recommended to adopt an exercise intensity of VT2 or above to enhance training responsiveness, which can be safely implemented using interval training (Wewege et al., 2018).

Older patients were not included in this study. As the degree to which exercise training adaptation is influenced by age-related impairments remains uncertain and aging itself is heterogeneous, the post-training responsiveness of CRF in older people might be complex (Erickson et al., 2023). Distinct from younger patients with MetS, older patients have typically developed cardiovascular diseases or have even experienced cardiac events, and the efficacy of the individualized method in enhancing training responsiveness within this group awaits verification (Hansen et al., 2019). Furthermore, numerous studies have demonstrated that various exercise modes and their combinations can effectively ameliorate multiple cardiometabolic indicators such as glycated hemoglobin, triglycerides, and C-reactive protein in diabetic patients (Batrakoulis et al., 2022a; Al-Mhanna et al., 2023; Al-Mhanna et al., 2024). Hence, apart from the training responsiveness of CRF, we should pay particular attention to the influence of each element of the exercise prescription on the training sensitivity of other health-related cardiometabolic indicators in the future.

There were some limitations to this study. We did not conduct a VO_2max_ verification test and used PeakVO_2_ as the primary marker, affecting the actual VO_2max_ response and actual VO_2max_ change in patients (Poole and Jones, 2017; Moreno-Cabañas et al., 2020; Weatherwax et al., 2018a). Although low-intensity adaptive training in Weeks 1–4 decreased the effects of subjective factors such as lack of exercise training experience and unfamiliarity with exercise methods, some patients might have achieved the minimum intensity threshold for increasing PeakVO_2_ during low-intensity adaptive training, decreasing the validity of the statistical results with respect to response. In addition, we did not perform a comprehensive follow-up of patients in this study, and the patients only verbally agreed not to alter their lifestyles and dietary habits during the intervention period. The International Physical Activity Questionnaire (IPAQ) was not used to determine the level of physical activity of the patients at baseline and post-intervention, which may have impacted the results of the present investigation. The absence of statistically significant differences between the groups might be attributed to the shorter intervention duration and the lower absolute exercise intensity. We did not anticipate that the initial emergence of these inter-group differences would be just right at the end of the study. Subsequent studies should continue to extend the intervention time to observe whether the differences will reach statistical significance. However, considering the importance of participant safety and compliance, the exercise plan and the study’s final outcomes have clinical value.

## 5 Conclusion

This study represents the first to compare the impact of two distinct exercise prescriptions, each with the same energy expenditure, on cardiopulmonary endurance, in individuals with MetS. Our findings reveal significant discrepancies in the condition of MetS patients when using the ventilatory threshold model, in contrast to the normal healthy population. Further, auxiliary analysis of CPET markers indicated that two patients experienced negative effects on their cardiopulmonary endurance following the exercise intervention, and these adverse changes were consistent across both cases. However, the underlying causes of this phenomenon remain unclear. These observations may contribute to the understanding of the varied responses in cardiopulmonary endurance post-training, often referred to as “non-response.” Additionally, the study shows that individualized exercise plans based on the ventilatory threshold model still yield unique benefits, even when the absolute exercise intensity is reduced.

## Data Availability

The original contributions presented in the study are included in the article/supplementary material, further inquiries can be directed to the corresponding author.

## References

[B1] Alberti K. G. Eckel R. H. Grundy S. M. Zimmet P. Z. Cleeman J. I. Donato K. A. (2009). Harmonizing the metabolic syndrome: a joint interim statement of the international diabetes federation task force on epidemiology and prevention; national heart, lung, and blood institute; American heart association; world heart federation; international atherosclerosis society; and international association for the study of obesity. Circulation 120, 1640–1645. 10.1161/CIRCULATIONAHA.109.192644 19805654

[B2] AL-Mhanna S. B. Batrakoulis A. Ghazali W. S. W. Mohamed M. Aldayel A. Alhussain M. H. (2024). Effects of combined aerobic and resistance training on glycemic control, blood pressure, inflammation, cardiorespiratory fitness and quality of life in patients with type 2 diabetes and overweight/obesity: a systematic review and meta-analysis. PeerJ 12, e17525. 10.7717/peerj.17525 38887616 PMC11182026

[B3] AL-Mhanna S. B. Rocha-Rodriguesc S. Mohamed M. Batrakoulis A. Aldhahi M. I. Afolabi H. A. (2023). Effects of combined aerobic exercise and diet on cardiometabolic health in patients with obesity and type 2 diabetes: a systematic review and meta-analysis. BMC Sports Sci. Med. Rehabilitation 15, 165. 10.1186/s13102-023-00766-5 PMC10696788 38049873

[B4] A'Naja M. N. Batrakoulis A. Camhi S. M. Mcavoy C. Sansone J. S. Reed R. (2024). 2025 ACSM worldwide fitness trends: future directions of the health and fitness industry. ACSM's Health and Fit. J. 28, 11–25. 10.1249/fit.0000000000001017

[B5] Astorino T. A. Emma D. (2021). Utility of verification testing to confirm attainment of maximal oxygen uptake in unhealthy participants: a perspective review. Sports 9, 108. 10.3390/sports9080108 34437369 PMC8402360

[B6] Bärtsch P. (1999). Platelet activation with exercise and risk of cardiac events. Lancet 354, 1747–1748. 10.1016/S0140-6736(99)90259-3 10577632

[B7] Batrakoulis A. Jamurtas A. Z. Fatouros I. G. (2021). High-intensity interval training in metabolic diseases: physiological adaptations. ACSM's Health and Fit. J. 25, 54–59. 10.1249/fit.0000000000000703

[B8] Batrakoulis A. Jamurtas A. Z. Fatouros I. G. (2022a). Exercise and type II diabetes mellitus: a brief guide for exercise professionals. Strength and Cond. J. 44, 64–72. 10.1519/ssc.0000000000000731

[B9] Batrakoulis A. Jamurtas A. Z. Metsios G. S. Perivoliotis K. Liguori G. Feito Y. (2022b). Comparative efficacy of 5 exercise types on cardiometabolic health in overweight and obese adults: a systematic review and network meta-analysis of 81 randomized controlled trials. Circulation Cardiovasc. Qual. Outcomes 15, e008243. 10.1161/CIRCOUTCOMES.121.008243 35477256

[B10] Bhati P. Shenoy S. Hussain M. E. (2018). Exercise training and cardiac autonomic function in type 2 diabetes mellitus: a systematic review. Diabetes and Metabolic Syndrome Clin. Res. and Rev. 12, 69–78. 10.1016/j.dsx.2017.08.015 28888482

[B11] Bhatti J. S. Bhatti G. K. Reddy P. H. (2017). Mitochondrial dysfunction and oxidative stress in metabolic disorders—a step towards mitochondria based therapeutic strategies. Biochimica Biophysica Acta (BBA)-Molecular Basis Dis. 1863, 1066–1077. 10.1016/j.bbadis.2016.11.010 PMC5423868 27836629

[B12] Binder R. K. Wonisch M. Corra U. Cohen-Solal A. Vanhees L. Saner H. (2008). Methodological approach to the first and second lactate threshold in incremental cardiopulmonary exercise testing. Eur. J. Prev. Cardiol. 15, 726–734. 10.1097/HJR.0b013e328304fed4 19050438

[B13] Bishop D. J. Botella J. Genders A. J. Lee M. J. Saner N. J. Kuang J. (2019). High-intensity exercise and mitochondrial biogenesis: current controversies and future research directions. Physiology 34, 56–70. 10.1152/physiol.00038.2018 30540234

[B14] Cadroy Y. Pillard F. Sakariassen K. S. Thalamas C. Boneu B. Riviere D. (2002). Strenuous but not moderate exercise increases the thrombotic tendency in healthy sedentary male volunteers. J. Appl. Physiology 93, 829–833. 10.1152/japplphysiol.00206.2002 12183474

[B15] Chan S. M. Selemidis S. Bozinovski S. Vlahos R. (2019). Pathobiological mechanisms underlying metabolic syndrome (MetS) in chronic obstructive pulmonary disease (COPD): clinical significance and therapeutic strategies. Pharmacol. and Ther. 198, 160–188. 10.1016/j.pharmthera.2019.02.013 30822464 PMC7112632

[B16] Chen J. Wang X. Dong B. Liu C. Zhao J. Dong Y. (2022). Cardiac function and exercise capacity in patients with metabolic syndrome: a cross-sectional study. Front. Cardiovasc. Med. 9, 974802. 10.3389/fcvm.2022.974802 36035938 PMC9410700

[B17] Cottin F. MéDIGUE C. LeprêTRE P.-M. Papelier Y. Koralsztein J.-P. Billat V. (2004). Heart rate variability during exercise performed below and above ventilatory threshold. Med. and Sci. Sports and Exerc. 36, 594–600. 10.1249/01.mss.0000121982.14718.2a 15064586

[B18] Dalleck L. C. Haney D. E. Buchanan C. A. Weatherwax R. M. (2016). Does a personalised exercise prescription enhance training efficacy and limit training unresponsiveness? A randomised controlled trial. J. Fit. Res. 5, 15–27.

[B19] Erickson M. L. Allen J. M. Beavers D. P. Collins L. M. Davidson K. W. Erickson K. I. (2023). Understanding heterogeneity of responses to, and optimizing clinical efficacy of, exercise training in older adults: NIH NIA Workshop summary. GeroScience 45, 569–589. 10.1007/s11357-022-00668-3 36242693 PMC9886780

[B20] Farah B. Ritti‐Dias R. Balagopal P. Hill J. Prado W. (2014). Does exercise intensity affect blood pressure and heart rate in obese adolescents? A 6‐month multidisciplinary randomized intervention study. Pediatr. Obes. 9, 111–120. 10.1111/j.2047-6310.2012.00145.x 23447453

[B21] Gaskill S. Walker A. Serfass R. Bouchard C. Gagnon J. Rao D. (2001). Changes in ventilatory threshold with exercise training in a sedentary population: the HERITAGE Family Study. Int. J. sports Med. 22, 586–592. 10.1055/s-2001-18522 11719894

[B22] Glaab T. Taube C. (2022). Practical guide to cardiopulmonary exercise testing in adults. Respir. Res. 23, 9–12. 10.1186/s12931-021-01895-6 35022059 PMC8754079

[B23] Green S. Askew C. (2018). V̇o2peak is an acceptable estimate of cardiorespiratory fitness but not V̇o2max. J. Appl. Physiology 125, 229–232. 10.1152/japplphysiol.00850.2017 29420148

[B24] Guio DE Prada V. Ortega J. F. Ramirez-Jimenez M. Morales-Palomo F. Pallares J. G. Mora-Rodriguez R. (2019). Training intensity relative to ventilatory thresholds determines cardiorespiratory fitness improvements in sedentary adults with obesity. Eur. J. Sport Sci. 19, 549–556. 10.1080/17461391.2018.1540659 30381027

[B25] Hackett D. A. (2020). Lung function and respiratory muscle adaptations of endurance-and strength-trained males. Sports 8, 160. 10.3390/sports8120160 33321800 PMC7764033

[B26] Hansen D. Abreu A. Ambrosetti M. Cornelissen V. Gevaert A. Kemps H. (2022). Exercise intensity assessment and prescription in cardiovascular rehabilitation and beyond: why and how: a position statement from the Secondary Prevention and Rehabilitation Section of the European Association of Preventive Cardiology. Eur. J. Prev. Cardiol. 29, 230–245. 10.1093/eurjpc/zwab007 34077542

[B27] Hansen D. Bonne K. Alders T. Hermans A. Copermans K. Swinnen H. (2019). Exercise training intensity determination in cardiovascular rehabilitation: should the guidelines be reconsidered? Eur. J. Prev. Cardiol. 26, 1921–1928. 10.1177/2047487319859450 31219704

[B28] Hansen D. Stevens A. Eijnde B. O. Dendale P. (2012). Endurance exercise intensity determination in the rehabilitation of coronary artery disease patients: a critical re-appraisal of current evidence. Sports Med. 42, 11–30. 10.2165/11595460-000000000-00000 22145810

[B29] Hopkins W. G. (2000). Measures of reliability in sports medicine and science. Sports Med. 30, 1–15. 10.2165/00007256-200030010-00001 10907753

[B30] Iannetta D. Inglis E. C. Mattu A. T. Fontana F. Y. Pogliaghi S. Keir D. A. (2020). A critical evaluation of current methods for exercise prescription in women and men. Med. Sci. Sports Exerc. 52, 466–473. 10.1249/MSS.0000000000002147 31479001

[B31] Jamnick N. A. Pettitt R. W. Granata C. Pyne D. B. Bishop D. J. (2020). An examination and critique of current methods to determine exercise intensity. Sports Med. 50, 1729–1756. 10.1007/s40279-020-01322-8 32729096

[B32] Kim B. Ku M. Kiyoji T. Isobe T. Sakae T. Oh S. (2020). Cardiorespiratory fitness is strongly linked to metabolic syndrome among physical fitness components: a retrospective cross-sectional study. J. Physiological Anthropol. 39, 30–39. 10.1186/s40101-020-00241-x PMC7528584 33004082

[B33] Liguori G. Medicine A. C. O. S. (2020). ACSM's guidelines for exercise testing and prescription. Lippincott Williams and Wilkins.

[B34] Macdonald T. L. Pattamaprapanont P. Pathak P. Fernandez N. Freitas E. C. Hafida S. (2020). Hyperglycaemia is associated with impaired muscle signalling and aerobic adaptation to exercise. Nat. Metab. 2, 902–917. 10.1038/s42255-020-0240-7 32694831 PMC8278496

[B35] Margaritelis N. V. Theodorou A. A. Paschalis V. Veskoukis A. S. Dipla K. Zafeiridis A. (2018). Adaptations to endurance training depend on exercise‐induced oxidative stress: exploiting redox interindividual variability. Acta Physiol. 222, e12898. 10.1111/apha.12898 28544643

[B36] Mattioni Maturana F. Soares R. N. Murias J. M. Schellhorn P. Erz G. Burgstahler C. (2021). Responders and non‐responders to aerobic exercise training: beyond the evaluation of. Physiol. Rep. 9, e14951. 10.14814/phy2.14951 34409753 PMC8374384

[B37] Meyler S. Bottoms L. Muniz‐Pumares D. (2021). Biological and methodological factors affecting response variability to endurance training and the influence of exercise intensity prescription. Exp. Physiol. 106, 1410–1424. 10.1113/EP089565 34036650

[B38] Mezzani A. Hamm L. F. Jones A. M. Mcbride P. E. Moholdt T. Stone J. A. (2013). Aerobic exercise intensity assessment and prescription in cardiac rehabilitation: a joint position statement of the European association for cardiovascular prevention and rehabilitation, the American association of cardiovascular and pulmonary rehabilitation and the Canadian association of cardiac rehabilitation. Eur. J. Prev. Cardiol. 20, 442–467. 10.1177/2047487312460484 23104970

[B39] Morales-Palomo F. Ramirez-Jimenez M. Ortega J. F. Moreno-CabañAS A. Mora-Rodriguez R. (2020). Exercise training adaptations in metabolic syndrome individuals on chronic statin treatment. J. Clin. Endocrinol. and Metabolism 105, dgz304–e1704. 10.1210/clinem/dgz304 31875915

[B40] Moreno‐CabañAS A. Ortega J. F. Morales‐Palomo F. Ramirez‐Jimenez M. Mora‐Rodriguez R. (2020). Importance of a verification test to accurately assess V̇O2 max in unfit individuals with obesity. Scand. J. Med. and Sci. Sports 30, 583–590. 10.1111/sms.13602 31746500

[B41] Passoni E. Lania A. Adamo S. Grasso G. S. Noè D. Miserocchi G. (2015). Mild training program in metabolic syndrome improves the efficiency of the oxygen pathway. Respir. physiology and Neurobiol. 208, 8–14. 10.1016/j.resp.2014.12.017 25554064

[B42] Pearson M. Smart N. (2018). Exercise therapy and autonomic function in heart failure patients: a systematic review and meta-analysis. Heart Fail. Rev. 23, 91–108. 10.1007/s10741-017-9662-z 29185161

[B43] Pichon A. (2007). Cardiovascular variability is/is not an index of autonomic control of circulation. J. Appl. Physiology 102, 503. 10.1152/japplphysiol.01038.2006 16990499

[B44] Poole D. C. Jones A. M. (2017). Measurement of the maximum oxygen uptake V̇o_2max_: V̇o_2peak_ is no longer acceptable. J. Appl. Physiology 122, 997–1002. 10.1152/japplphysiol.01063.2016 28153947

[B45] Pymer S. Nichols S. Prosser J. Birkett S. Carroll S. Ingle L. (2020). Does exercise prescription based on estimated heart rate training zones exceed the ventilatory anaerobic threshold in patients with coronary heart disease undergoing usual-care cardiovascular rehabilitation? A United Kingdom perspective. Eur. J. Prev. Cardiol. 27, 579–589. 10.1177/2047487319852711 31116574

[B46] Rodriguez J. C. Peterman J. E. Fleenor B. S. Whaley M. H. Kaminsky L. A. Harber M. P. (2022). Cardiopulmonary exercise responses in individuals with metabolic syndrome: the ball state adult fitness longitudinal lifestyle study. Metabolic Syndrome Relat. Disord. 20, 414–420. 10.1089/met.2021.0130 35527641

[B47] Sarmiento S. GarcíA-Manso J. M. MartíN-GonzáLEZ J. M. Vaamonde D. CalderóN J. Da Silva-Grigoletto M. E. (2013). Heart rate variability during high-intensity exercise. J. Syst. Sci. Complex. 26, 104–116. 10.1007/s11424-013-2287-y

[B48] Scharhag‐Rosenberger F. Walitzek S. Kindermann W. Meyer T. (2012). Differences in adaptations to 1 year of aerobic endurance training: individual patterns of nonresponse. Scand. J. Med. and Sci. Sports 22, 113–118. 10.1111/j.1600-0838.2010.01139.x 20561283

[B49] Silva L. R. ZamunéR A. R. Gentil P. Alves F. M. Leal A. G. Soares V. (2017). Cardiac autonomic modulation and the kinetics of heart rate responses in the on-and off-transient during exercise in women with metabolic syndrome. Front. Physiology 8, 542. 10.3389/fphys.2017.00542 PMC5526966 28798697

[B50] Skinner J. S. Jaskólski A. Jaskólska A. Krasnoff J. Gagnon J. Leon A. S. (2001). Age, sex, race, initial fitness, and response to training: the HERITAGE Family Study. J. Appl. Physiology 90, 1770–1776. 10.1152/jappl.2001.90.5.1770 11299267

[B51] Stuckey M. I. Tulppo M. P. Kiviniemi A. M. Petrella R. J. (2014). Heart rate variability and the metabolic syndrome: a systematic review of the literature. Diabetes/metabolism Res. Rev. 30, 784–793. 10.1002/dmrr.2555 24816921

[B52] Tirandi A. Carbone F. Montecucco F. Liberale L. (2022). The role of metabolic syndrome in sudden cardiac death risk: recent evidence and future directions. Eur. J. Clin. Investigation 52, e13693. 10.1111/eci.13693 PMC9286662 34714544

[B53] Weatherwax R. M. Harris N. K. Kilding A. E. Dalleck L. C. (2016). The incidence of training responsiveness to cardiorespiratory fitness and cardiometabolic measurements following individualized and standardized exercise prescription: study protocol for a randomized controlled trial. Trials 17, 601–612. 10.1186/s13063-016-1735-0 27993169 PMC5168814

[B54] Weatherwax R. M. Harris N. K. Kilding A. E. Dalleck L. C. (2018a). Using a site-specific technical error to establish training responsiveness: a preliminary explorative study. Open Access J. Sports Med. 9, 47–53. 10.2147/OAJSM.S155440 29563845 PMC5848661

[B55] Weatherwax R. M. Harris N. K. Kilding A. E. Dalleck L. C. (2019). Incidence of VO2max responders to personalized versus standardized exercise prescription. Med. Sci. Sports Exerc 51, 681–691. 10.1249/MSS.0000000000001842 30673687

[B56] Weatherwax R. M. Ramos J. S. Harris N. K. Kilding A. E. Dalleck L. C. (2018b). Changes in metabolic syndrome severity following individualized versus standardized exercise prescription: a feasibility study. Int. J. Environ. Res. public Health 15, 2594. 10.3390/ijerph15112594 30463388 PMC6265765

[B57] Wewege M. A. Ahn D. Yu J. Liou K. Keech A. (2018). High‐intensity interval training for patients with cardiovascular disease—is it safe? A systematic review. J. Am. Heart Assoc. 7, e009305. 10.1161/JAHA.118.009305 30376749 PMC6404189

[B58] Willson C. Watanabe M. Tsuji‐Hosokawa A. Makino A. (2019). Pulmonary vascular dysfunction in metabolic syndrome. J. Physiology 597, 1121–1141. 10.1113/JP275856 PMC6375868 30125956

[B59] Wolpern A. E. Burgos D. J. Janot J. M. Dalleck L. C. (2015). Is a threshold-based model a superior method to the relative percent concept for establishing individual exercise intensity? a randomized controlled trial. BMC sports Sci. Med. rehabilitation 7, 16–19. 10.1186/s13102-015-0011-z PMC4491229 26146564

